# Cartilage Warping in Rhinoplasty: An Assessment of Temperature, Carving, and Suturing Conditions

**DOI:** 10.1093/asjof/ojae007.036

**Published:** 2024-04-12

**Authors:** Yasmine Ibrahim, Sumun Khetpal, Wayne Ozaki, Jason Roostaeian

**Affiliations:** University of California - Los Angeles, Los Angeles; University of California-Los Angeles, Los Angeles, CA; University of California - Los Angeles, Los Angeles; David Geffen School of Medicine at UCLA, Los Angeles, CA

## Abstract

**Goals/Purpose:**

Rhinoplasty is often contingent on the successful harvesting and allocation of cartilage in order to build the nasal framework. While autologous and cadaveric cartilage grafts have been utilized, it remains unclear how factors, such as suturing and carving techniques, as well as temperature, impact its short- and long-term warping potential. Our systematic review seeks to objectively assess how various carving (i.e. peripheral versus central, eccentric versus concentric), suture patterns (i.e. counter-balancing, oppositional), and temperature conditions impact the warping potential of cartilage grafts in rhinoplasty. In conducting this study, we hope to determine the optimal conditions for stable cartilage constructs in rhinoplasty.

**Methods/Technique:**

A systematic literature review was conducted using a combination of terms including, “warp,” “rhinoplasty,” “cartilage,” “suture,” “temperature,” “carving,” and “cutting.” Removal of duplicates was performed, followed by further screening by abstract and full text. To reduce the possibility of bias, a standardized form was used for data extraction; variables including author, year of publication, journal of publication, study design, conditions, and results were recorded.

**Results/Complications:**

A total of eleven studies were included in the analysis. Cartilage types included fresh frozen cadaveric graft, autologous costal cartilage, and bovine rib cartilage. Conditions associated with reduced warping include central and concentric based carving patterns, oppositional suturing; Peripherally and eccentric cut cartilage, as well as increased time, were associated with a higher degree of warping.

**Conclusion:**

To the authors’ knowledge, our systematic analysis is the first to summarize the impact of various conditions on cartilage warping in rhinoplasty. Moreover, we determine the protective nature of certain carving and suturing methods - particularly central, concentric carving, and oppositional suturing - in minimizing cartilage warping. With this information, we hope to guide plastic surgeons in optimizing conditions to potentially prevent cartilage warping, and ultimately, provide reliable and sustainable results for patients undergoing rhinoplasty.

